# Hypomagnesemia in adults with type 2 diabetes mellitus in Riyadh, Saudi Arabia: A cross-sectional study

**DOI:** 10.1097/MD.0000000000041253

**Published:** 2025-01-17

**Authors:** Nasser M. Al-Daghri, Sobhy M. Yakout, Syed Danish Hussain, Abdullah M. Alnaami, Nicola Veronese, Mario Barbagallo, Shaun Sabico

**Affiliations:** a Department of Biochemistry, College of Science, King Saud University, Riyadh, Kingdom of Saudi Arabia; b Department of Health Promotion, Mother and Child Care, Internal Medicine and Medical Specialties, University of Palermo, Palermo, Italy.

**Keywords:** hypomagnesemia, magnesium deficiency, metabolic profile, Riyadh, Saudi Arabia, type 2 diabetes mellitus

## Abstract

This study investigates the prevalence of hypomagnesemia in adults with type 2 diabetes mellitus (T2DM) in Riyadh, Saudi Arabia, and examines its association with various metabolic parameters. Conducted as a cross-sectional study at King Saud University, Riyadh, it included 294 Saudi adults aged 25 to 65 years, comprising 119 T2DM patients, 80 prediabetics, and 95 nondiabetic controls. Participants underwent physical examinations, and fasting blood samples were analyzed for glucose, glycated hemoglobin (HbA1c), lipid profile, and serum magnesium levels. Statistical analysis revealed that lower magnesium levels were significantly more prevalent in T2DM patients (1.65 ± 4.9 mg/L) compared to prediabetes (2.48 ± 5.2 mg/L) and controls (2.9 ± 5.4 mg/L; *P* < .001). T2DM patients with magnesium deficiency exhibited higher levels of fasting glucose (11.2 ± 3.9 mmol/L), HbA1c (8.6 ± 2.1 mmol/L), and triglycerides (2.1 ± 0.9 mmol/L), along with increased insulin resistance (Homeostatic Model Assessment of Insulin Resistance = 6.6) and decreased insulin sensitivity (Quantitative Insulin Sensitivity Check Index = 0.29). Magnesium levels correlated negatively with glucose (R = −0.58) and HbA1c (R = −0.61). The area under the curve for serum magnesium in predicting HbA1c > 5.7 was 0.88, and for HbA1c ≥ 6.5, it was 0.91, indicating high diagnostic accuracy. These findings suggest that magnesium deficiency significantly impacts the metabolic profile of T2DM patients in Riyadh. Therefore, routine monitoring of magnesium levels is crucial in diabetes management, and further research is needed to explore the benefits of magnesium supplementation in T2DM care.

## 1. Introduction

Magnesium (Mg), the second most abundant intracellular cation after potassium, is crucial for human health. It is a key cofactor in over 300 enzymatic reactions, influencing a broad spectrum of biochemical functions in the body.^[1]^ The role of Mg is particularly significant in glucose metabolism and the modulation of smooth muscle function, rendering it an essential micronutrient.^[2]^ The deficiency of Mg, notably in conditions such as Type 2 diabetes mellitus (T2DM), has been linked to various clinical challenges.^[3]^

Recent studies have highlighted the influence of Mg on insulin receptor activity, insulin-mediated glucose uptake, and its potential to improve insulin sensitivity, all of which underscore the importance of maintaining adequate Mg levels.^[4,5]^ Moreover, the anti-inflammatory and antioxidative properties of Mg have been shown to play a role in mitigating metabolic complications, further emphasizing its therapeutic relevance in the context of T2DM.^[6]^

With diabetes mellitus on the rise globally, it has become a major health concern, especially in low- and middle-income countries. According to the World Health Organization, more than 150 million people aged over 20 years were living with diabetes in 2000, a figure projected to increase significantly.^[7]^ By 2035, the International Diabetes Federation estimates that nearly 592 million people will be affected by diabetes, emphasizing its prevalence as a major noncommunicable disease and a leading cause of death in the developed world.^[8,9]^ Factors like obesity, lack of physical activity, and aging populations are driving this increase. This rising trend is particularly pronounced in low- and middle-income countries, where rapid urbanization, sedentary lifestyles, and dietary changes have contributed to increasing rates of obesity and diabetes. Recent data indicates that the prevalence of T2DM has been accelerating faster in these regions compared to high-income countries, highlighting an urgent need for effective management and prevention strategies.^[9]^

In the Kingdom of Saudi Arabia, the prevalence of diabetes is among the highest worldwide, with estimates ranging from 20% to 35%.^[10,11]^ This situation not only presents significant public health challenges but also imposes a heavy economic burden on healthcare systems due to the costs associated with diabetes care. The increasing trend of diabetes in Saudi Arabia mirrors the global pattern, underlining the urgency for proactive measures to combat this escalating health crisis.

Dietary habits, lifestyle factors, and genetic predispositions in the Saudi population may contribute to the higher prevalence of T2DM and related conditions such as hypomagnesemia.^[12]^ Studies have suggested that traditional diets low in Mg, along with a shift towards more processed and Westernized food consumption, could exacerbate micronutrient deficiencies, making Mg a key area of focus for diabetes management in this region.^[13]^

The importance of micronutrients and trace elements, such as Mg, in the management and development of diabetes is increasingly acknowledged.^[14,15]^ Mg plays a fundamental role in glucose metabolism and insulin sensitivity, with studies linking low Mg levels to impaired insulin secretion and action, thereby increasing the risk of T2DM.^[15,16]^ Mg supplementation has been proposed to improve insulin sensitivity and metabolic control in diabetic patients, highlighting its potential therapeutic benefits.^[17]^ This accentuates the need to consider nutritional factors, including Mg levels, in comprehensive diabetes management strategies.

Several interventional studies have demonstrated that Mg supplementation can lead to improvements in glycemic control, insulin resistance, and lipid profiles in individuals with T2DM.^[14,18]^ These findings suggest a possible therapeutic role for Mg, not just in preventing the onset of diabetes but also in managing its progression and complications.^[14,19]^

Epidemiological evidence consistently shows a relationship between hypomagnesemia and a higher prevalence of T2DM, with a significant inverse correlation between Mg intake and T2DM risk.^[15,20,21]^ This relationship exhibits a linear dose-response pattern, suggesting that increased Mg intake may reduce T2DM risk. Mg deficiency is implicated in glucose intolerance and insulin resistance, potentially leading to poor glycemic control and an increased risk of long-term diabetes complications.^[22,23]^

Despite this evidence, there is a lack of localized research addressing the prevalence of hypomagnesemia in specific populations such as those in Saudi Arabia. Previous studies conducted in other Middle Eastern countries have reported similar patterns, indicating that regional lifestyle and dietary factors could be significant determinants of micronutrient deficiencies.^[24,25]^

The mechanism linking diabetes and Mg deficiency, while not fully understood, indicates a bidirectional relationship where diabetes can lead to hypomagnesemia, which in turn may exacerbate the condition.^[26,27]^ This study aims to investigate the prevalence of hypomagnesemia in adults with T2DM in Riyadh, Saudi Arabia, and its implications for diabetes management. By exploring the role of Mg, the study seeks to enhance the understanding and development of more effective treatment and prevention strategies for this widespread condition. Furthermore, this research intends to fill existing gaps in literature by providing data specific to the Saudi population, thereby contributing to global efforts in understanding the complex interactions between micronutrient status and diabetes.

## 2. Methodology

### 2.1. Subjects selection

In this cross-sectional study, 294 Saudi adults aged 25 to 65 years were examined, including individuals with T2DM (119 participants), prediabetes (Pre-DM, 80 participants), and nondiabetic controls (95 participants). Participant data were sourced from a comprehensive database at the Chair for Biomarkers of Chronic Diseases at King Saud University, Riyadh. This database compiles clinical information from individuals involved in various diabetes prevention initiatives across primary healthcare centers. The classification of diabetes and prediabetes was in line with the World Health Organization guidelines, categorizing individuals with fasting blood glucose levels above 7.0 mmol/L as diabetic and those with levels between 6.1 and 7.0 mmol/L as prediabetic.^[28]^ The study was conducted under the approval of the Ethics Committee of the College of Science Research Center, King Saud University (approval No. 8/25/454239), ensuring ethical compliance and participant consent. All subjects provided written informed consent and completed a comprehensive questionnaire that included demographic information and medical history. Physical examinations were conducted to exclude any participants with critical health conditions such as cardiac, kidney, or liver diseases; mental health issues; or those on specific medications. Key health metrics measured included body mass index (BMI, kg/m^2^), waist-hip ratio (WHR), and blood pressure (systolic and diastolic, mm Hg), ensuring a thorough assessment of each participant’s health status. It is important to note that T2DM patients who were on glucose-lowering medications were included in the T2DM group regardless of their fasting blood glucose levels at the time of measurement. This was done to ensure that all patients diagnosed with T2DM were correctly categorized, even if their medication effectively lowered their fasting glucose levels below 7.0 mmol/L. Healthy controls were individuals without a history of diabetes or prediabetes, confirmed by fasting glucose levels consistently below 6.1 mmol/L. Prediabetic individuals were classified based on fasting glucose levels between 6.1 and 7.0 mmol/L, following the World Health Organization guidelines.

### 2.2. Biochemical analysis

Fasting blood samples were collected from each participant. These were then processed, aliquoted, and transported to the Chair for Biomarkers of Chronic Diseases laboratory for detailed biochemical analysis. The fasting glucose levels and lipid profile, including total cholesterol, high-density lipoprotein (HDL)-cholesterol, and triglycerides, were measured using the Konelab 20XT chemical analyzer (Thermo Scientific, Vantaa, Finland) and standard bioassay kits. The levels of glycated hemoglobin (HbA1c) were determined using the D-10 Hemoglobin Analyzer (Bio-Rad Laboratories, Hercules), a system that employs an ion-exchange high-performance liquid chromatography method for accurate measurement. Serum insulin was determined using the LIAISON XL automated quantitative analyzer (DiaSorin, Saluggia, Italy). It uses an advanced chemiluminescence technique with magnetic microparticle separation to achieve the best sensitivity and accuracy of the assay.

For the assessment of insulin resistance, the Homeostatic Model Assessment (HOMA) and the Quantitative Insulin Sensitivity Check Index (QUICKI) were calculated using the formulas:

HOMA = (fasting insulin [µIU/mL] × fasting glucose [mg/dL])/405, and QUICKI = 1/(log[fasting insulin µIU/mL] + log[fasting glucose mg/dL]).

Insulin resistance was defined as a HOMA value of ≥ 2.6 and a QUICKI value of ≤ 0.337, aligning with established clinical thresholds.^[29–31]^

### 2.3. Mg determination

In this procedure, 150 µL of nitric acid was combined with 150 µL of the sample and 100 µL of hydrogen peroxide in Eppendorf tubes. These tubes were then centrifuged at a speed of 4400 rpm for 10 minutes at a temperature of 4°C. Following centrifugation, the tubes were placed in a block digester and heated at 95°C for 90 minutes. After digestion, the volume in each tube was brought up to 1 mL with distilled water and the tubes were stored at 4°C. For the analysis, 250 µL of each prepared sample was diluted to 3 mL using deionized water. The quantification of Mg was conducted using an Inductively Coupled Plasma-Mass Spectrometer, specifically the NexION 300 D model from Perkin Elmer.

Considering the ongoing debate and varying international standards regarding the optimal cutoff serum concentration for Mg deficiency, our study acknowledges the complexity of establishing a universally accepted threshold. Notably, different countries and research groups have proposed varying cutoff points. In Russia and some other countries, a commonly used lower reference limit is 0.66 mmol/L.^[4]^ However, extensive historical data in the United States identified 0.75 mmol/L as the reference limit, with recent publications suggesting that the cutoff should be higher, at 0.8 mmol/L.^[32–34]^ Moreover, 2 groups of researchers, 1 in the United States^[33]^ and 1 in Germany,^[35]^ have independently suggested a serum Mg value of 0.85 mmol/L as the low cutoff point to define hypomagnesemia. They argue that serum Mg values below 0.85 mmol/L (2.07 mg/dL) are associated with an increased risk of various diseases, including cardiovascular disease, metabolic conditions, obesity, and implications related to aging.

Given these diverse perspectives and the evidence presented, our study opts to adopt an evidence-based reference interval, considering serum Mg values < 0.8 mmol/L as indicative of Mg deficiency. This decision aligns with the more recent and conservative approaches in the literature, aiming to more accurately identify individuals at risk of cardiovascular disease, T2DM, and other related diseases.^[33,36,37]^ We believe this cutoff provides a pragmatic balance between the various international standards and the emerging consensus on the implications of lower Mg levels.

### 2.4. Statistical analysis

In this study, data analysis was conducted using Statistical Package for the Social Sciences, version 21.0. Continuous variables were described using mean and standard deviations if normally distributed and median values (spanning from the first to the third quartile) for those not normally distributed. Categorical data were presented in terms of frequencies and percentages. For assessing significant differences in mean values of normally distributed variables among groups, ANOVA was employed, while the Kruskal-Wallis test was utilized for evaluating significant median differences in non-normal variables. Bonferroni correction was used to adjust for multiple comparisons. The Chi-square test was applied to analyze differences in proportions among categorical variables. Linear correlations between select variables were determined using the Pearson correlation coefficient for variables having a normal distribution. All non-normal variables underwent a log transformation before any parametric tests were conducted. Statistical significance was set at a *P* < .05.

## 3. Results

The study included 294 participants (mean age 50.9 years, 59% female, 41% male), categorized into 3 groups: control (n = 95), prediabetes (n = 80), and diabetes (n = 119). Table 1 summarizes the descriptive statistics of the groups, focusing on various health parameters. The results revealed a statistically significant progression in mean age across the groups, with the control group at 40.9 ± 13.7 years, prediabetes at 46.7 ± 10.9 years, and diabetes at 52.2 ± 11.2 years (*P* < .001). BMI was also higher in the prediabetes (32.6 ± 7.6 kg/m²) and diabetes groups (31.8 ± 6.0 kg/m²) compared to the control group (29.9 ± 6.0 kg/m², *P* = .017). WHR showed an upward trend from the control and prediabetes groups (0.9 ± 0.1) to the diabetes group (1.0 ± 0.1, *P* = .003). Systolic blood pressure significantly increased in the diabetes group (128.5 ± 15.1 mm Hg) compared to the control group (112.3 ± 10.3 mm Hg, *P* < .001), and a similar pattern was observed for diastolic blood pressure (diabetes: 79.6 ± 9.0 mm Hg, control: 74.1 ± 7.0 mm Hg, *P* = .004). Both glucose and HbA1c levels showed a notable rise across the groups, peaking in the diabetes group (glucose: 11.8 ± 3.3 mmol/L, HbA1c: 8.9 ± 1.9, *P* < .001 for both). Total cholesterol levels were higher in the prediabetes (5.1 ± 1.1 mmol/L) and diabetes groups (5.0 ± 1.2 mmol/L) compared to the control group (4.6 ± 1.1 mmol/L, *P* = .008), with triglyceride levels significantly elevated in the diabetes group (2.1 ± 0.9 mmol/L, *P* < .001).

**Table 1:** Descriptive statistics according to diabetes status of patients.

Parameters	Control	Prediabetes	Diabetes	*P* value	*P* value*
N	95	80	119	--	--
Age (yr)	40.9 ± 13.7	46.7 ± 10.9	52.2 ± 11.2†‡	0.000	--
Female/male	68/27	49/31	57/62	0.002	--
BMI (kg/m^2^)	29.9 ± 6.0	32.6 ± 7.6	31.8 ± 6.0	0.017	--
WHR	0.9 ± 0.1	0.9 ± 0.1	1.0 ± 0.1	0.003	0.132
Systolic BP (mm Hg)	112.3 ± 10.3	124.3 ± 14.1†	128.5 ± 15.1†	<0.001	<0.001
Diastolic BP (mm Hg)	74.1 ± 7.0	78.5 ± 8.0	79.6 ± 9.0	0.004	0.060
Glucose (mmol/L)	5.4 ± 0.6	6.1 ± 0.4	11.8 ± 3.3†‡	<0.001	<0.001
HbA1c	4.8 ± 0.8	6.0 ± 0.2†	8.9 ± 1.9†‡	<0.001	<0.001
Insulin (uU/mL)	12.8 (8.0–18.9)	15.2 (10.1–22.3)	14.8 (10.2–22.5)	0.079	0.348
Total cholesterol (mmol/L)	4.6 ± 1.1	5.1 ± 1.1†	5.0 ± 1.2†	0.008	0.026
HDL-cholesterol (mmol/L)	1.0 ± 0.4	0.9 ± 0.3	0.9 ± 0.3	0.734	0.912
LDL-cholesterol (mmol/L)	3.0 ± 1.0	3.2 ± 1.0	2.9 ± 1.0	0.111	0.197
Triglycerides (mmol/L)	1.5 ± 0.7	2.0 ± 0.9†	2.1 ± 0.9†	<0.001	0.001
Albumin (g/L)	39.6 ± 5.1	40.4 ± 5.3	41.5 ± 4.8	0.029	0.483
Calcium (mmol/L)	2.3 ± 0.2	2.4 ± 0.2	2.5 ± 0.2†‡	<0.001	<0.001
Magnesium (mg/dL)	2.9 ± 5.4	2.48 ± 5.2†	1.65 ± 4.9†‡	<0.001	<0.001

Data presented as mean ± SD for normal variables while median (1st quartile–3rd quartile) is presented for non-normal variables; *P* values are obtained from 1-way analysis of variance (ANOVA); all non-normal variables were log-transformed prior to parametric testing. BMI = body mass index, BP = blood pressure, HbA1c = glycated hemoglobin, HDL = high-density lipoprotein, LDL = low-density lipoprotein, WHR = waist-hip ratio.

* *P*-value adjusted age, BMI and gender; *P* < .05 considered significant. Boneforonni correction was applied to adjust for multiple comparisons.

† Statistically significant difference (*P* < .05) between the respective group and the control group.

‡ Statistically significant difference (*P* < .05) between the respective group and the prediabetes group.

**Figure 1.**
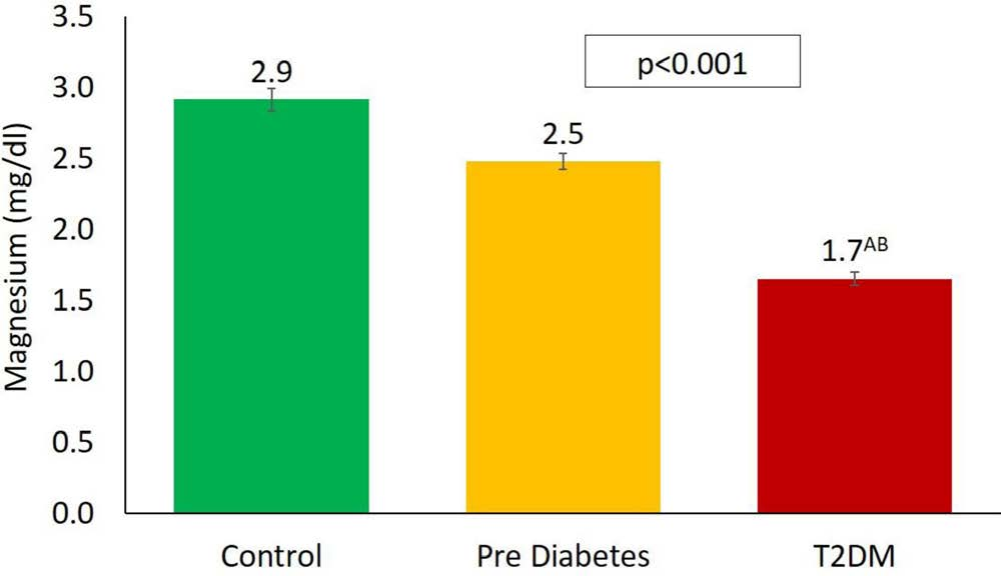
Magnesium levels according to diabetic status. (A) Significant compared to control (B) Prediabetes. T2DM = type 2 diabetes mellitus.

Additionally, the study observed that patients with T2DM had significantly lower mean serum Mg levels than both prediabetes patients and healthy controls, suggesting a possible link between Mg levels and the progression from prediabetes to type 2 diabetes. Figure 1 visually compares serum Mg levels across these groups, clearly highlighting the significant differences. When adjusting the data for age using the general linear model, no significant changes were observed across the control, prediabetes, and diabetes groups.

**Figure 2.**
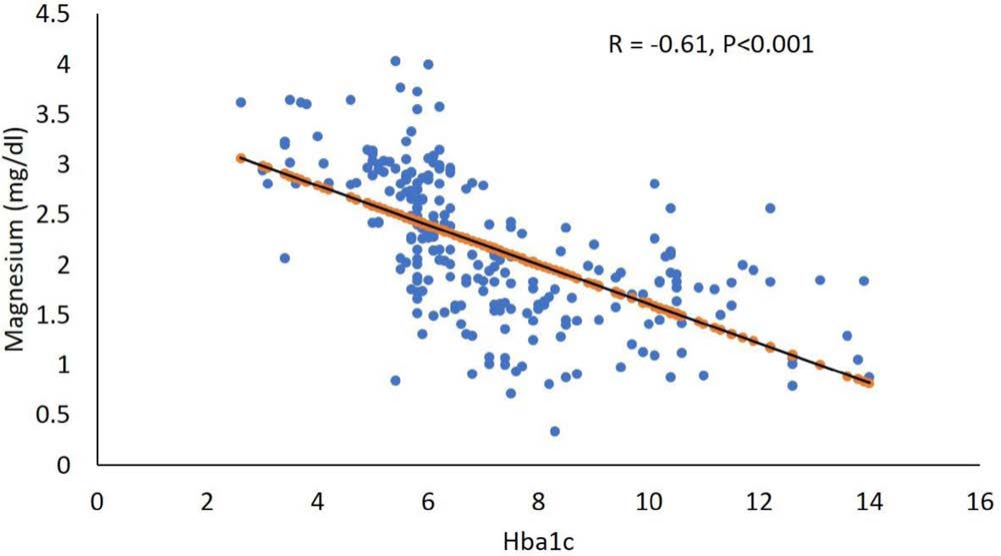
Correlation between glycated hemoglobin (Hba1c) and magnesium (mg/dL) in all patients.

Table 2 presents a more detailed comparison of health parameters between individuals categorized as Mg nondeficient (>1.944 mg/dL) and deficient (<1.944 mg/dL). The study included 142 nondeficient and 101 Mg-deficient individuals. The Mg-deficient group exhibited markedly higher glucose (11.2 ± 3.9 mmol/L) and HbA1c levels (8.6 ± 2.1, *P* < .001 for both), indicating a strong association between Mg deficiency and impaired glucose metabolism. Furthermore, this group had higher insulin resistance (Homeostatic Model Assessment of Insulin Resistance [HOMA-IR], *P* < .001) and lower insulin sensitivity (QUICKI, *P* < .001). Triglycerides were significantly higher (*P* = .015), and calcium levels were also elevated (*P* = .002) in the deficient group. Other parameters, including blood pressure, total cholesterol, HDL, low-density lipoprotein-cholesterol, and albumin levels, did not show significant differences. These findings underscore the critical impact of Mg deficiency on key health indicators, particularly those related to glucose metabolism and insulin sensitivity. The correlation between serum Mg levels and HbA1c, as depicted in Figure 2, further illustrates this link, emphasizing the strong negative correlation between these parameters.

**Table 2:** Descriptive statistics according to magnesium deficiency.

	Nondeficient (>1.944 mg/dL)	Deficient (<1.944 mg/dL)	*P* value
N	142	101	
Age (yr)	46.8 ± 12.6	51.7 ± 10.9	0.002
BMI (kg/m^2^)	31.7 ± 6.6	32.2 ± 6.4	0.610
Male/female	51/91	48/53	0.070
WHR	0.9 ± 0.1	1.0 ± 0.1	0.087
Systolic BP (mm Hg)	125.1 ± 15.6	127.4 ± 14.3	0.330
Diastolic BP (mm Hg)	79.2 ± 8.4	78.8 ± 8.6	0.736
Glucose (mmol/L)	6.9 ± 2.5	11.2 ± 3.9	<0.001
HbA1c	6.1 ± 1.7	8.6 ± 2.1	<0.001
Insulin (uU/mL)*	14.7 (8.9–19.8)	13.9 (10.3–21.1)	0.556
HOMA-IR	4.3 (2.6–7.5)	6.6 (4.8–10.9)	<0.001
QUICKI	0.31 (0.29–0.33)	0.29 (0.27–0.30)	<0.001
Total cholesterol (mmol/L)	4.9 ± 1.1	5.0 ± 1.2	0.474
HDL-cholesterol (mmol/L)	0.9 ± 0.3	1.0 ± 0.3	0.230
LDL-cholesterol (mmol/L)	3.1 ± 0.9	2.9 ± 1.0	0.295
Triglycerides (mmol/L)	1.8 ± 0.9	2.1 ± 0.9	0.015
Albumin (g/L)	40.3 ± 5.0	41.1 ± 4.8	0.244
Calcium (mmol/L)	2.4 ± 0.2	2.5 ± 0.2	0.002
Magnesium (mg/dL)	2.7 ± 0.5	1.5 ± 0.4	<0.001

Data presented as mean ± SD for normal variables while median (1st quartile–3rd quartile) is presented for non-normal variables; *P* values are obtained from independent sample *t* test; all non-normal variables were log-transformed prior to parametric testing; *P* < .05 considered significant. BMI = body mass index, BP = blood pressure, HbA1c = glycated hemoglobin, HDL = high-density lipoprotein, HOMA-IR = Homeostatic Model Assessment of Insulin Resistance, LDL = low-density lipoprotein, QUICKI = Quantitative Insulin Sensitivity Check Index, WHR = waist-hip ratio.

* Indicates non-normal variables.

Table 3 compares Mg deficiency prevalence among individuals with prediabetes and type 2 diabetes. It shows a markedly higher prevalence of Mg deficiency in the diabetes group, with 76% (88 out of 116) being deficient, compared to only 15% (12 out of 79) in the prediabetes group. This suggests that Mg may play a significant role in the progression or management of diabetes. Although most health parameters, including age, BMI, gender ratio, WHR, blood pressure, HbA1c, insulin levels, HOMA-IR, vitamin D, albumin, and calcium, showed no significant differences in relation to Mg levels, 2 exceptions were noted. First, Mg-deficient individuals in the diabetes group had a near-significant higher glucose level (*P* = .077), suggesting a potential link between Mg deficiency and glucose control. Second, there was a significant difference in HDL-cholesterol levels, where Mg-deficient individuals had notably higher levels (*P* = .04), indicating a possible relationship between Mg deficiency and altered lipid profiles in diabetic patients.

**Table 3:** Descriptive statistics according to prediabetes and diabetes status.

	Prediabetes			Diabetes		
	Mg nondeficient	Mg deficient	*P* value	Mg nondeficient	Mg deficient	*P* value
N	67 (84.8)	12 (15.2)		28 (24.1)	88 (75.9)	
Age (yr)	46.9 ± 11.1	46.7 ± 10.0	0.717	50.6 ± 13.9	52.7 ± 10.7	0.815
BMI (kg/m^2^)	33.1 ± 8.0	31.0 ± 6.1	0.42	30.1 ± 4.5	32.2 ± 6.3	0.132
Male/female	27/40	3/9	0.315	15/13	45/43	0.822
WHR	0.9 ± 0.1	1.0 ± 0.1	0.276	1.0 ± 0.1	1.0 ± 0.1	0.383
Systolic BP (mm Hg)	123.9 ± 14.6	130.7 ± 1.6	0.288	130.5 ± 15.0	127.6 ± 14.7	0.213
Diastolic BP (mm Hg)	78.6 ± 7.1	81.0 ± 10.5	0.519	83.5 ± 9.3	78.5 ± 8.4	0.082
Glucose (mmol/L)	6.1 ± 0.4	6.0 ± 0.4	0.796	11.1 ± 2.7	12.0 ± 3.4	0.077
HbA1c	6.0 ± 0.2	6.0 ± 0.2	0.869	8.5 ± 1.6	9.0 ± 2.0	0.204
Insulin (uU/mL)*	15.7 (10.4–20.9)	13.8 (6.6–26.8)	0.735	17.7 (11.3–27.6)	13.9 (10.1–20.8)	0.728
HOMA-IR	4.4 (2.7–6.0)	3.6 (1.7–7.0)	0.764	9.9 (5.1–12.8)	6.6 (4.8–11.8)	0.684
QUICKI	0.3 (0.3–0.3)	0.3 (0.3–0.4)	0.577	0.3 (0.3–0.3)	0.3 (0.3–0.3)	0.921
Total cholesterol (mmol/L)	5.2 ± 1.1	4.8 ± 1.2	0.18	5.3 ± 1.1	5.0 ± 1.2	0.952
HDL-cholesterol (mmol/L)	1.0 ± 0.3	0.8 ± 0.2	0.179	0.8 ± 0.3	1.0 ± 0.3	0.04
LDL-cholesterol (mmol/L)	3.3 ± 1.0	3.0 ± 1.0	0.218	3.3 ± 1.1	2.9 ± 0.9	0.694
Triglycerides (mmol/L)	1.9 ± 0.8	2.1 ± 1.1	0.434	2.1 ± 1.1	2.1 ± 0.9	0.951
Vitamin D (nmol/L)*	39.7 (27.8–68.5)	56.4 (35.6–87.8)	0.569	54.3 (50.3–58.4)	50.9 (38.4–62.0)	0.136
Albumin (g/L)	40.9 ± 5.7	38.6 ± 3.1	0.124	42.0 ± 4.6	41.4 ± 4.8	0.772
Calcium (mmol/L)	2.4 ± 0.2	2.3 ± 0.2	0.112	2.5 ± 0.2	2.5 ± 0.2	0.575
Magnesium (mg/L)	2.7 ± 0.4	1.8 ± 0.2	<0.001	2.4 ± 0.3	1.5 ± 0.4	<0.001

Data presented as mean ± SD for normal variables while median (1st quartile–3rd quartile) is presented for non-normal variables; *P* values are obtained from independent sample *t* test; all non-normal variables were log-transformed prior to parametric testing; *P* < .05 considered significant. In this table, “A” indicates a statistically significant difference (*P* < .05) between the respective group and the control group, while “B” indicates a statistically significant difference (*P* < .05) between the respective group and the prediabetes group. BMI = body mass index, BP = blood pressure, HbA1c = glycated hemoglobin, HDL = high-density lipoprotein, HOMA-IR = Homeostatic Model Assessment of Insulin Resistance, LDL = low-density lipoprotein, QUICKI = Quantitative Insulin Sensitivity Check Index, WHR = waist-hip ratio.

* Indicates non-normal variables.

Table 4 provides an analysis of linear correlations between Mg levels and various health parameters. Across the overall group, lower Mg levels were strongly correlated with higher glucose (R = −0.58) and HbA1c levels (R = −0.61; Fig. 2), as well as increased insulin resistance (HOMA-IR: R = −0.28) and decreased insulin sensitivity (QUICKI: R = 0.30). In the Mg nondeficient group, a notable negative correlation was observed with systolic blood pressure (R = −0.26). Conversely, in the Mg-deficient group, positive correlations with age (R = 0.23) and albumin (R = 0.20) were noted. Linear regression analysis showed that an increase of 1 unit in Mg levels is linked to a 0.15-unit decrease in HbA1c (adjusted R-square = 29.4%, *P* < .001). These findings emphasize the importance of Mg in maintaining metabolic health, particularly in regulating blood glucose and insulin function. Figure 2 effectively visualizes this correlation, clearly demonstrating the association between Mg levels and HbA1c.

**Table 4:** Correlation between magnesium and select parameters.

	Overall		Mg nondeficient		Mg deficient	
	R	*P*	R	*P*	R	*P*
Age (yr)	−0.14	0.026	−0.06	0.458	0.23	0.020
BMI (kg/m^2^)	−0.01	0.870	0.13	0.126	−0.17	0.096
WHR	−0.05	0.664	0.21	0.157	0.19	0.235
Systolic BP (mm Hg)	−0.14	0.073	−0.26	0.028	−0.01	0.905
Diastolic BP (mm Hg)	−0.01	0.899	−0.20	0.098	0.07	0.534
Glucose (mmol/L)	−0.58	0.000	−0.41	0.000	−0.07	0.475
HbA1c	−0.61	0.000	−0.49	0.000	−0.12	0.224
Insulin (uU/mL)*	−0.02	0.792	−0.07	0.530	0.18	0.104
HOMA-IR	−0.28	0.000	−0.25	0.022	0.12	0.273
QUICKI	0.30	0.000	0.25	0.018	−0.12	0.286
Total cholesterol (mmol/L)	−0.09	0.182	−0.15	0.072	0.03	0.783
HDL-cholesterol (mmol/L)	−0.06	0.379	0.07	0.431	−0.12	0.287
LDL-cholesterol (mmol/L)	0.04	0.545	−0.09	0.323	0.02	0.833
Triglycerides (mmol/L)	−0.18	0.006	−0.19	0.028	0.08	0.440
Vitamin D (nmol/L)*	0.07	0.295	0.03	0.749	0.02	0.851
Albumin (g/L)	−0.02	0.769	0.00	0.964	0.20	0.045
Calcium (mmol/L)	−0.21	0.001	−0.03	0.759	−0.18	0.076

Data presented as Pearson correlation coefficient (R); *P* < .05 considered significant In this table, “A” indicates a statistically significant difference (*P* < .05) between the respective group and the control group, while “B” indicates a statistically significant difference (*P* < .05) between the respective group and the prediabetes group. BMI = body mass index, BP = blood pressure, HbA1c = glycated hemoglobin, HDL = high-density lipoprotein, HOMA-IR = Homeostatic Model Assessment of Insulin Resistance, LDL = low-density lipoprotein, QUICKI = Quantitative Insulin Sensitivity Check Index, WHR = waist-hip ratio.

* Indicates non-normal variables.

**Figure 3.**
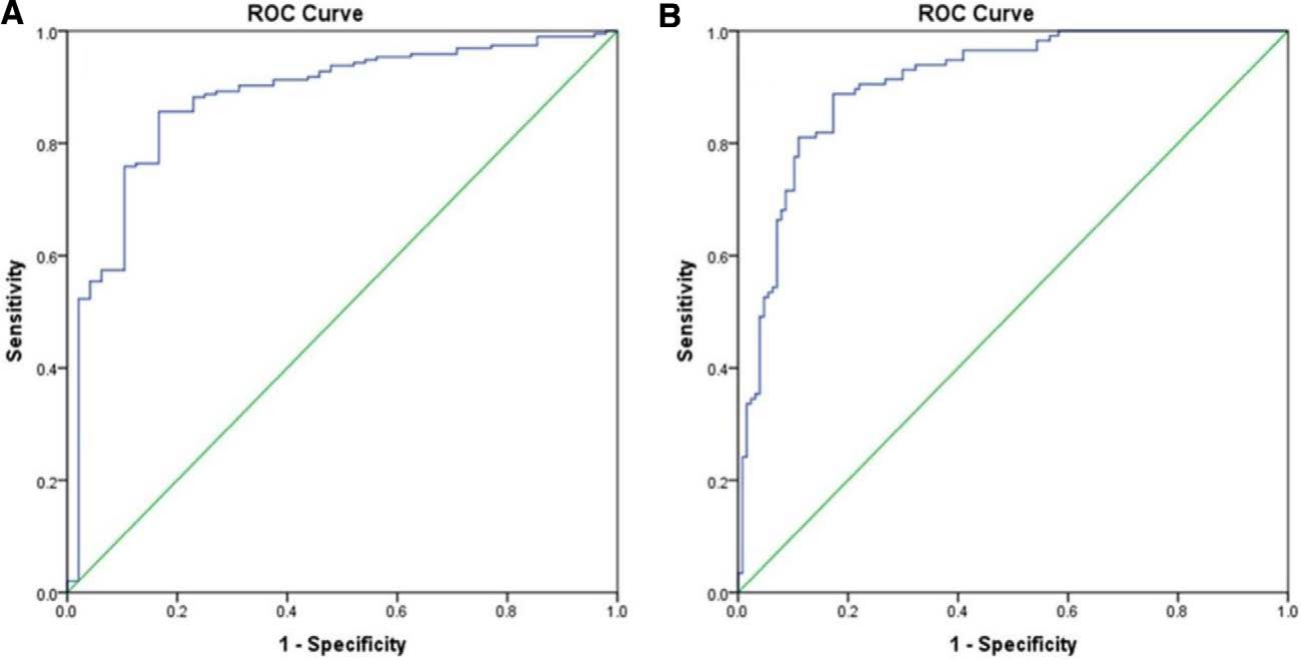
Receiver operating characteristic (ROC) curve of diagnosing abnormal glycated hemoglobin (Hba1c) (A) > 5.6 and (B) ≥ 6.5.

In the context of this study on Mg deficiency and its potential role in predicting T2DM, the ROC analysis presented in Figure 3 and Table 5 yields substantial findings. With an area under the curve (AUC) of 0.88 for HbA1c > 5.7 and 0.91 for HbA1c ≥ 6.5, both with *P* values < 0.001 (Fig. 3), the data indicate strong link between Mg deficiency and abnormal glycemic levels associated with T2DM. These results suggest that serum Mg levels could serve as a predictive biomarker for diabetes, reinforcing the necessity for routine monitoring and management of Mg status in individuals at risk for, or already diagnosed with, T2DM. The high accuracy of the AUC values underscores the potential for integrating Mg level assessments into standard diabetes screening and management protocols.

**Table 5:** Area under the curve for diagnosing abnormal Hba1c.

	AUC ± SE	Cutoff (mg/dL)	Sensitivity	Specificity	*P* value
Abnormal Hba1c > 5.7	0.88 ± 0.03	2.56	80.00%	83.30%	<0.001
T2DM (Hba1c ≥ 6.5)	0.91 ± 0.02	2.00	80.20%	88.90%	<0.001

Data presented as area under the curve (AUC) obtained from receiver operating characteristic analysis. *P* < .05 considered significant. In this table, “A” indicates a statistically significant difference (*P* < .05) between the respective group and the control group, while “B” indicates a statistically significant difference (*P* < .05) between the respective group and the prediabetes group. HbA1c = glycated hemoglobin, SE = standard error, T2DM = type 2 diabetes mellitus.

## 4. Discussions

This study, conducted in Riyadh, Saudi Arabia, provides crucial insights into the prevalence of hypomagnesemia among adults with T2DM and its implications on various health parameters. Our findings reveal a significant association between Mg deficiency and the exacerbation of T2DM, which is consistent with the growing body of evidence linking hypomagnesemia to poor metabolic control in diabetic patients.^[2,3,38,39]^ Specifically, our results showed that T2DM patients with hypomagnesemia had significantly higher fasting glucose and HbA1c levels compared to those with normal Mg levels. This finding underscores the role of Mg in glucose metabolism, where Mg deficiency can impair insulin-mediated glucose uptake, exacerbating hyperglycemia and increasing the risk of complications.^[15,23,40,41]^

The physiological mechanism behind these observations may involve Mg’s role as a cofactor in several enzymatic reactions critical for insulin action. Mg is essential for the activation of tyrosine kinase in the insulin receptor, which is necessary for glucose uptake.^[3]^ Consequently, a deficiency in Mg can reduce the phosphorylation of the insulin receptor, impair insulin signaling, and contribute to hyperglycemia. This mechanism explains the strong negative correlation observed in our study between Mg levels and both glucose and HbA1c levels.^[16,41]^

Interestingly, despite the strong association between hypomagnesemia and poor glycemic control, our study did not find significant differences in fasting glucose levels between the Mg-deficient and nondeficient groups among T2DM subjects. This could be attributed to the use of glucose-lowering medications by many T2DM patients, which may have masked the potential impact of Mg deficiency on fasting glucose levels. However, the significant differences observed in HbA1c levels suggest that long-term glycemic control is more adversely affected by Mg deficiency, reflecting its cumulative impact over time.^[1,11,42]^

In addition to glycemic control, our study also found that hypomagnesemia was associated with increased insulin resistance, as evidenced by higher HOMA-IR values and lower QUICKI scores in Mg-deficient individuals. This supports the hypothesis that Mg deficiency contributes to insulin resistance, a key factor in the pathogenesis of T2DM. Mg is known to influence insulin receptor tyrosine kinase activity and postreceptor signaling pathways, which are crucial for insulin sensitivity.^[42–44]^ Our findings align with previous studies that have demonstrated the role of Mg in improving insulin sensitivity and reducing insulin resistance.^[39,45]^

Furthermore, our study found no significant differences in blood pressure and lipid profiles between Mg-deficient and nondeficient groups. However, a noteworthy observation was the negative correlation between Mg levels and systolic blood pressure in nondeficient individuals. This finding is consistent with existing literature that suggests adequate Mg levels contribute to vascular smooth muscle relaxation and lower blood pressure, potentially acting as a natural calcium channel blocker.^[30,46]^ The absence of significant changes in lipid profiles could be due to the complex interplay between Mg and lipid metabolism, which requires further investigation.^[32,47]^

Another important aspect of our study is the relationship between Mg deficiency and age. We found a positive correlation between age and the prevalence of hypomagnesemia among T2DM patients, indicating that older adults are at a higher risk of Mg deficiency. This could be due to age-related changes in Mg metabolism, such as reduced dietary intake, altered intestinal absorption, and increased renal loss of Mg.^[15,33]^ These findings suggest that elderly diabetic patients may particularly benefit from regular monitoring of Mg levels and appropriate dietary supplementation.

Moreover, the elevated calcium levels observed in Mg-deficient individuals in our study suggest a disruption in calcium-Mg homeostasis. Mg acts as a natural antagonist to calcium, and its deficiency can lead to intracellular calcium overload, which has been implicated in insulin resistance and hypertension.^[42,48]^ This supports the theory that Mg deficiency might alter calcium metabolism and disrupt the delicate balance between these 2 essential minerals, potentially contributing to metabolic and cardiovascular complications in T2DM patients.

Our study also highlighted the potential clinical utility of serum Mg levels as a biomarker for poor glycemic control in T2DM patients. The AUC values for predicting abnormal HbA1c levels were notably high, indicating that Mg deficiency is strongly associated with poor glycemic outcomes. These findings suggest that routine assessment of Mg levels could enhance diabetes management strategies by identifying patients at risk of poor glycemic control and guiding interventions such as Mg supplementation.^[22,26,49]^

The strengths of our study include its focus on a specific population in Riyadh, Saudi Arabia, where the prevalence of T2DM is among the highest globally. The comprehensive biochemical analysis of glucose metabolism markers, insulin resistance indices, and Mg levels provides a robust dataset for examining the complex interactions between these variables. Additionally, our study adheres to strict WHO guidelines for diabetes classification, enhancing the reliability of our findings.

While this study provides valuable insights, there are some limitations to consider. The cross-sectional design restricts our ability to establish causality between Mg deficiency and T2DM outcomes, necessitating longitudinal studies to clarify this relationship. Additionally, focusing on a single geographic region may limit the generalizability of the findings to populations with different dietary habits and genetic backgrounds. The study also lacked detailed data on the use of glucose-lowering medications and did not account for other confounding factors, such as dietary intake of Mg, physical activity, and coexisting micronutrient deficiencies. Furthermore, a single time-point measurement of serum Mg may not accurately reflect long-term status. Future research should aim to address these aspects to provide a more comprehensive understanding of the role of Mg in diabetes.

## 5. Conclusion

This study highlights a significant association between Mg deficiency and poor glycemic control in adults with T2DM in Riyadh, Saudi Arabia. Lower serum Mg levels were strongly correlated with higher glucose and HbA1c levels, suggesting that Mg deficiency may exacerbate insulin resistance and impaired glucose metabolism. The findings indicate that routine monitoring of Mg levels could be a valuable addition to diabetes management strategies, potentially improving glycemic outcomes. Further research is warranted to explore the benefits of Mg supplementation as part of comprehensive diabetes care. These results suggest that addressing Mg deficiency could enhance glycemic control and overall metabolic health in diabetic patients.

## Acknowledgments

This work was supported by Researchers Supporting Project number (RSP2024R21), King Saud University, Riyadh, Saudi Arabia.

## Author contributions

**Conceptualization:** Nasser Al-Daghri.

**Funding acquisition:** Nasser Al-Daghri.

**Writing – review & editing:** Nasser Al-Daghri, Syed Danish Hussain, Abdullah M. Alnaami, Nicola Veronese, Mario Barbagallo, Shaun Sabico.

**Methodology:** Sobhy Yakout, Abdullah M. Alnaami.

**Writing – original draft:** Sobhy Yakout.

**Formal analysis:** Syed Danish Hussain.

**Investigation:** Abdullah M. Alnaami.

**Supervision:** Nicola Veronese, Mario Barbagallo, Shaun Sabico.

**Validation:** Nicola Veronese, Mario Barbagallo, Shaun Sabico.

## References

[1] Swaminathan R. Magnesium metabolism and its disorders. Clin Biochem Rev. 2003;24:47–66. 18568054 PMC1855626

[2] Barbagallo M Dominguez LJ. Magnesium and type 2 diabetes. World J Diabetes. 2015;6:1152–7. 26322160 10.4239/wjd.v6.i10.1152 PMC4549665

[3] Pham PC Pham PM Pham SV Miller JM Pham PT. Hypomagnesemia in patients with type 2 diabetes. Clin J Am Soc Nephrol. 2007;2:366–73. 17699436 10.2215/CJN.02960906

[4] Yang SJ Hwang SY Baik SH. Serum magnesium level is associated with type 2 diabetes in women with a history of gestational diabetes mellitus: the Korea national diabetes program study. J Korean Med Sci. 2014;29:84–9. 24431910 10.3346/jkms.2014.29.1.84 PMC3890481

[5] Barbagallo M Dominguez LJ. Magnesium and aging. Curr Pharm Des. 2010;16:832–9. 20388094 10.2174/138161210790883679

[6] Guerrero-Romero F Tamez-Perez HE González-González G. Oral magnesium supplementation improves insulin sensitivity in non-diabetic subjects with insulin resistance. A double-blind placebo-controlled randomized trial. Diabetes Metab. 2004;30:253–8. 15223977 10.1016/s1262-3636(07)70116-7

[7] World Health Organization. Diabetes Mellitus Fact Sheet. 2003.

[8] Zimmet P Alberti KG Shaw J. Global and societal implications of the diabetes epidemic. Nature. 2001;414:782–7. 11742409 10.1038/414782a

[9] Cho NH Shaw JE Karuranga S. IDF diabetes atlas: global estimates of diabetes prevalence for 2017 and projections for 2045. Diabetes Res Clin Pract. 2018;138:271–81. 29496507 10.1016/j.diabres.2018.02.023

[10] Al-Nozha MM Al-Maatouq MA Al-Mazrou YY. Diabetes mellitus in Saudi Arabia. Saudi Med J. 2004;25:1603–10. 15573186

[11] Al Dawish MA Robert AA Braham R. Diabetes mellitus in Saudi Arabia: a review of the recent literature. Curr Diabetes Rev. 2016;12:359–68. 26206092 10.2174/1573399811666150724095130

[12] Hamarshih M Hamshari S Nazzal Z. Hypomagnesemia and poor glycemic control among type 2 diabetic patients: a cross-sectional study. Indian J Endocrinol Metab. 2022;26:575–80. 39005513 10.4103/ijem.ijem_213_22 PMC11245293

[13] Al-Ghamdi SM Cameron EC Sutton RA. Magnesium deficiency: pathophysiologic and clinical overview. Am J Kidney Dis. 1994;24:737–52. 7977315 10.1016/s0272-6386(12)80667-6

[14] Kumar SR Kumar KGS Gayathri R. Hypomagnesemia in patients with type 2 diabetes mellitus. J Assoc Physicians India. 2024;72:25–8. 10.59556/japi.72.0410 38990583

[15] Pitliya A Vasudevan SS Batra V. Global prevalence of hypomagnesemia in type 2 diabetes mellitus - a comprehensive systematic review and meta-analysis of observational studies. Endocrine. 2024;84:842–51. 38159172 10.1007/s12020-023-03670-7

[16] Guerrero-Romero F Rodriguez-Moran M. Low serum magnesium levels and metabolic syndrome. Acta Diabetol. 2002;39:209–13. 12486495 10.1007/s005920200036

[17] Larsson SC Wolk A. Magnesium intake and risk of type 2 diabetes: a meta-analysis. J Intern Med. 2007;262:208–14. 17645588 10.1111/j.1365-2796.2007.01840.x

[18] Mooren FC Krüger K Völker K Golf SW Wadepuhl M Kraus A. Oral magnesium supplementation reduces insulin resistance in non-diabetic subjects - a double-blind, placebo-controlled, randomized trial. Diabetes Obes Metab. 2011;13:281–4. 21205110 10.1111/j.1463-1326.2010.01332.x

[19] Sales CH Pedrosa Lde F. Magnesium and diabetes mellitus: their relation. Clin Nutr. 2006;25:554–62. 16690176 10.1016/j.clnu.2006.03.003

[20] Lopez-Ridaura R Willett WC Rimm EB. Magnesium intake and risk of type 2 diabetes in men and women. Diabetes Care. 2004;27:134–40. 14693979 10.2337/diacare.27.1.134

[21] Hruby A Meigs JB O’Donnell CJ Jacques PF McKeown NM. Higher magnesium intake reduces risk of impaired glucose and insulin metabolism and progression from prediabetes to diabetes in middle-aged americans. Diabetes Care. 2014;37:419–27. 24089547 10.2337/dc13-1397 PMC3898748

[22] Huerta MG Roemmich JN Kington ML. Magnesium deficiency is associated with insulin resistance in obese children. Diabetes Care. 2005;28:1175–81. 15855585 10.2337/diacare.28.5.1175

[23] Gommers LM Hoenderop JG Bindels RJ De Baaij JH. Hypomagnesemia in type 2 diabetes: a vicious circle? Diabetes. 2016;65:3–13. 26696633 10.2337/db15-1028

[24] Hoteit M Mortada H Al-Jawaldeh A; The Regional CORONA COOKING Survey Group. Dietary diversity in the Eastern Mediterranean region before and during the COVID-19 pandemic: disparities, challenges, and mitigation measures. Front Nutr. 2022;9:813154. 35252299 10.3389/fnut.2022.813154 PMC8893198

[25] Santos C Santos BDC De Carvalho GB. Magnesium status and dietary patterns associated with glycemic control in individuals with type 2 diabetes mellitus. Biol Trace Elem Res. 2023;201:5152–61. 36807884 10.1007/s12011-023-03601-7

[26] Fang X Han H Li M. Dose-response relationship between dietary magnesium intake and risk of type 2 diabetes mellitus: a systematic review and meta-regression analysis of prospective cohort studies. Nutrients. 2016;8:739. 27869762 10.3390/nu8110739 PMC5133122

[27] Kostov K. Effects of magnesium deficiency on mechanisms of insulin resistance in type 2 diabetes: focusing on the processes of insulin secretion and signaling. Int J Mol Sci. 2019;20:1351. 30889804 10.3390/ijms20061351 PMC6470576

[28] WHO. World Health Organization, International DF. Definition and Diagnosis of Diabetes Mellitus and Intermediate Hyperglycaemia: Report of a WHO/IDF Consultation. Geneva: World Health Organization; 2006.

[29] Himsworth HP. Diabetes mellitus: its differentiation into insulin-sensitive and insulin-insensitive types. Diabet Med. 2011;28:1440–4. 22092505 10.1111/j.1464-5491.2011.3508.x

[30] McAuley KA Williams SM Mann JI. Diagnosing insulin resistance in the general population. Diabetes Care. 2001;24:460–4. 11289468 10.2337/diacare.24.3.460

[31] Matsuda M DeFronzo RA. Insulin sensitivity indices obtained from oral glucose tolerance testing: comparison with the euglycemic insulin clamp. Diabetes Care. 1999;22:1462–70. 10480510 10.2337/diacare.22.9.1462

[32] Elin RJ. Assessment of magnesium status for diagnosis and therapy. Magnes Res. 2010;23:S194–198. 20736141 10.1684/mrh.2010.0213

[33] Costello RB Elin RJ Rosanoff A. Perspective: the case for an evidence-based reference interval for serum magnesium: the time has come. Adv Nutr. 2016;7:977–93. 28140318 10.3945/an.116.012765 PMC5105038

[34] Lowenstein FW Stanton MF. Serum magnesium levels in the United States, 1971-1974. J Am Coll Nutr. 1986;5:399–414. 3771947 10.1080/07315724.1986.10720143

[35] Micke O Vormann J Kraus A Kisters K. Serum magnesium: time for a standardized and evidence-based reference range. Magnes Res. 2021;34:84–9. 34463286 10.1684/mrh.2021.0486

[36] Orlova S Dikke G Pickering G Konchits S Starostin K Bevz A. Magnesium deficiency questionnaire: a new non-invasive magnesium deficiency screening tool developed using real-world data from four observational studies. Nutrients. 2020;12:2062. 32664490 10.3390/nu12072062 PMC7400907

[37] Rosanoff A West C Elin RJ; MaGNet Global Magnesium Project (MaGNet). Recommendation on an updated standardization of serum magnesium reference ranges. Eur J Nutr. 2022;61:3697–706. 35689124 10.1007/s00394-022-02916-w PMC9186275

[38] Zhao Y Zhou M Shang Y. Effects of co-supplementation of chromium and magnesium on metabolic profiles, inflammation, and oxidative stress in impaired glucose tolerance. Diab Vasc Dis Res. 2024;21:14791641241228156. 38228168 10.1177/14791641241228156 PMC10798099

[39] Gheorghe A-M Ciobica M-L Nistor C. Inquiry of the metabolic traits in relationship with daily magnesium intake: focus on type 2 diabetic population. Clin Pract. 2024;14:1319–47. 39051301 10.3390/clinpract14040107 PMC11270223

[40] Barbagallo M Dominguez LJ Galioto A. Role of magnesium in insulin action, diabetes and cardio-metabolic syndrome X. Mol Aspects Med. 2003;24:39–52. 12537988 10.1016/s0098-2997(02)00090-0

[41] Luo B Pan B Zhao G Li J Sun L. Association between serum magnesium levels and glycemic control in type 2 diabetes. Diabetes Metab Syndr Obes. 2024;17:2823–9. 39081371 10.2147/DMSO.S471787 PMC11288356

[42] Shahmoradi S Chiti H Tavakolizadeh M Hatami R Motamed N Ghaemi M. The effect of magnesium supplementation on insulin resistance and metabolic profiles in women with polycystic ovary syndrome: a randomized clinical trial. Biol Trace Elem Res. 2024;202:941–6. 37393389 10.1007/s12011-023-03744-7

[43] Houston M. The role of magnesium in hypertension and cardiovascular disease. J Clin Hypertens (Greenwich). 2011;13:843–7. 22051430 10.1111/j.1751-7176.2011.00538.x PMC8108907

[44] Shah CV Sparks MA Lee C-T. Sodium/glucose cotransporter 2 inhibitors and magnesium homeostasis: a review. Am J kidney Dis. 2024;83:648–58. 38372686 10.1053/j.ajkd.2023.11.006

[45] Kass L Weekes J Carpenter L. Effect of magnesium supplementation on blood pressure: a meta-analysis. Eur J Clin Nutr. 2012;66:411–8. 22318649 10.1038/ejcn.2012.4

[46] Xu M-R Wang A-P Wang Y-J Lu J-X Shen L Li L-X. Serum magnesium levels are negatively associated with obesity and abdominal obesity in type 2 diabetes mellitus: a real-world study. Korean Diabetes J. 2024;48:1147–59. 10.4093/dmj.2023.0401 PMC11621656 38807276

[47] Luo L Zhang Y Wang H Chen D Li L. The efficacy of magnesium supplementation for gestational diabetes: a meta-analysis of randomized controlled trials. Eur J Obstet Gynecol Reprod Biol. 2024;293:84–90. 38128389 10.1016/j.ejogrb.2023.12.014

[48] Maier JA. Low magnesium and atherosclerosis: an evidence-based link. Mol Aspects Med. 2003;24:137–46. 12537993 10.1016/s0098-2997(02)00095-x

[49] Drenthen LCA De Baaij JHF Rodwell L Van Herwaarden AE Tack CJ De Galan BE. Oral magnesium supplementation does not affect insulin sensitivity in people with insulin-treated type 2 diabetes and a low serum magnesium: a randomised controlled trial. Diabetologia. 2024;67:52–61. 37922013 10.1007/s00125-023-06029-9 PMC10709477

